# The Complete Response of a Renal Cell Carcinoma Metastatic to Brain, Lungs, and Liver to Second-Line Nivolumab: A Case Report

**DOI:** 10.7759/cureus.29680

**Published:** 2022-09-28

**Authors:** Mahsa Haghpanah, Armin Azimi, Pedram Fadavi, Seyed Morteza Bagheri, Amir Mohammad Arefpour

**Affiliations:** 1 Department of Radiation Oncology, Iran University of Medical Sciences, Tehran, IRN; 2 Department of Neurosurgery, Iran University of Medical Sciences, Tehran, IRN; 3 Department of Radiology, Hasheminejad Kidney Center, Iran University of Medical Sciences, Tehran, IRN; 4 Department of Radiation Oncology, Hasheminejad Kidney Center, Iran University of Medical Sciences, Tehran, IRN

**Keywords:** multiple metastases, complete response, abscopal effect, nivolumab, immune checkpoint inhibitor, metastatic renal cell carcinoma

## Abstract

Metastatic renal cell carcinoma (RCC) is a therapeutic challenge to clinicians since it shows significant resistance to chemotherapy and radiation therapy. With the introduction of immunotherapy, the treatment paradigm for RCC has evolved. Here, we describe the case of a 55-year-old male who presented with flank pain. An abdominal-pelvic computed tomography (CT) scan revealed a right renal mass. Following open right radical nephrectomy, first-line treatment with sunitinib was administered. After four months he developed multiple metastases to the liver, lungs, abdominal wall, and brain. He initiated second-line treatment with nivolumab and also received whole brain radiation therapy (WBRT). Six months following combined treatment with nivolumab and WBRT, a CT scan revealed complete radiologic response in the lungs, abdominal wall, brain, and liver except for the persistence of a subhepatic mass. Despite the discontinuation of nivolumab and starting bevacizumab due to financial problems, the patient was stable for 22 months, and after this, he was hospitalized with high bilirubin levels. An abdominal CT scan detected the development of the necrotic subhepatic mass compressing the common bile duct (CBD), with no other sign of metastatic disease. We believe that the explanation for this long-term disease control could be the combination of immune-checkpoint-inhibitor (ICI) with WBRT resulting in significant cranial and extracranial immune response, known as "the abscopal effect". This report highlights the importance of local therapy combined with ICI-based therapy in metastatic RCC.

## Introduction

Renal cell carcinoma (RCC) accounts for 3% of adult malignancies, approximately 338,000 new cases are diagnosed with RCC worldwide each year, and almost 30% of patients present with metastatic disease at the time of diagnosis [1]. Nivolumab is a fully-humanized monoclonal immunoglobulin-G4 programmed death 1 (PD-1) checkpoint inhibitor which was approved for the treatment of advanced RCC previously treated with antiangiogenic therapy, based on overall survival (OS) benefit observed on Checkmate 025 trial [2].

According to the literature, there is no evidence to support the efficacy of any immune checkpoint inhibitor (ICI) based therapies in patients with RCC metastatic to the brain, as most studies excluded patients with brain metastases. The rationale for this exclusion is the increased size of ICIs which limits their ability to cross the blood-tumor barrier [3,4]. Here we present the case of a patient with clear cell type RCC metastatic to the brain, lungs, and liver which showed complete radiologic response to second-line nivolumab.

## Case presentation

**Figure 1:**
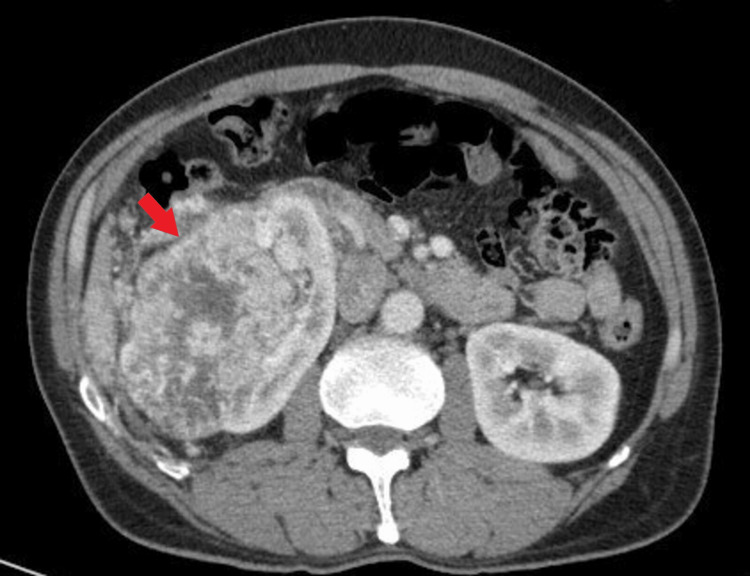
Initial CT scan of the abdomen and pelvis The red arrow is showing a large heterogenous right renal mass.

A 55-year-old heavy smoker male with no relevant medical history presented in September 2019 with flank pain and significant weight loss. Subsequent computed tomography (CT) scan revealed a right renal mass measuring 12.4×9 cm with no other pathologic findings (Figure 1).

Baseline lab data were unremarkable. Per International Metastatic RCC Database Consortium (IMDC) criteria, his disease was intermediate-risk at the time of diagnosis and his Karnofsky performance status (KPS) was 70%.

**Figure 2:**
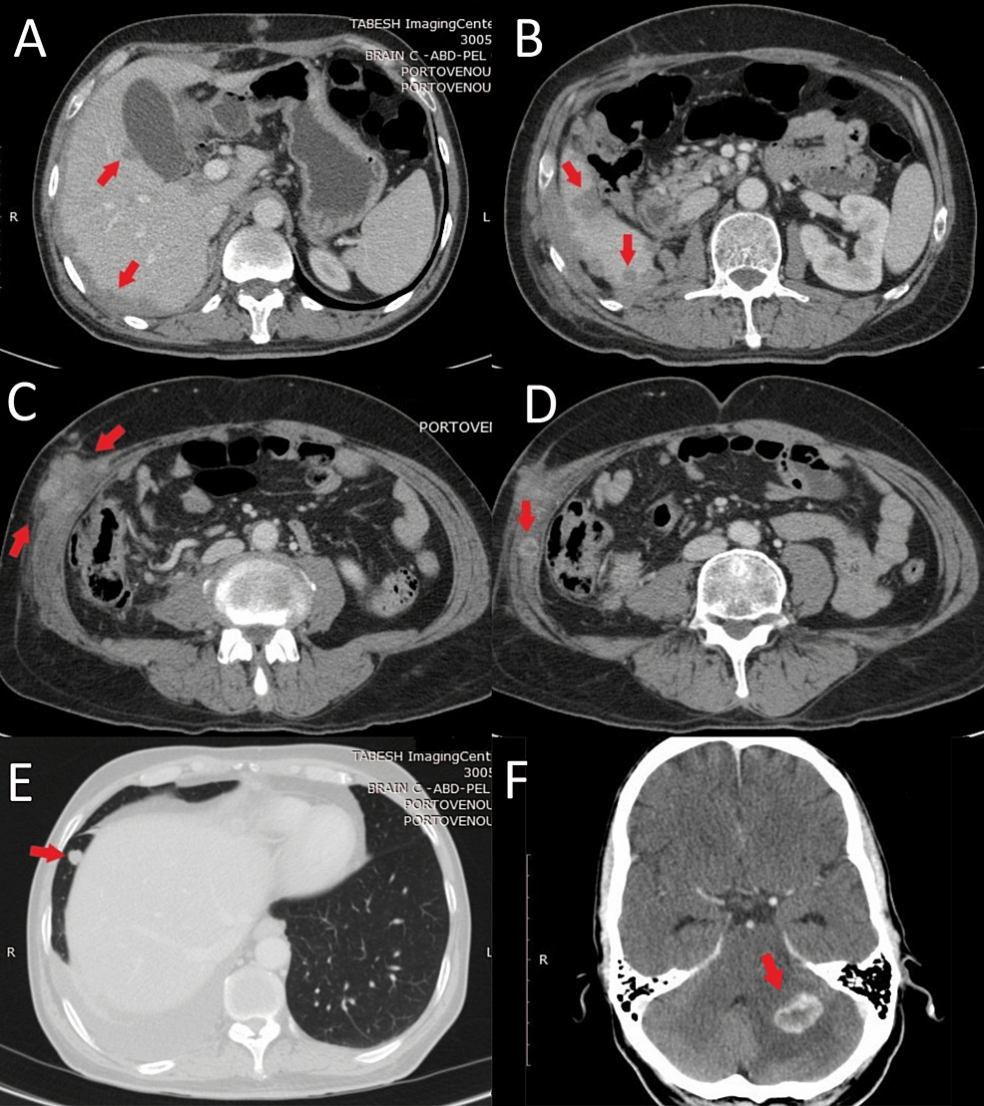
CT images for multiple metastatic lesions Multiple metastatic lesions to the (A, B) liver, (C, D) abdominal wall, (E) lungs, and (F) brain after four months of treatment with sunitinib.

Open right radical nephrectomy was performed in November 2019 and the histopathologic diagnosis revealed clear cell type RCC with massive necrosis and no sarcomatoid differentiation along with invasion to the renal pelvis, adrenal gland, omental fat, and diaphragm. Accordingly, the patient was commenced on adjuvant sunitinib 50 mg on the 3/1 schedule (50 mg once daily for three consecutive weeks on treatment followed by one-week-off). Following four months of treatment with sunitinib, the patient became icteric and developed a progressive headache. Further evaluations discovered multiple masses in the renal fossa and abdominal wall, multiple round lesions in the right hepatic lobe, and a few right pleural-based nodules. He was also found to have two enhancing lesions (20×15 mm and 5×3 mm) in the posterior fossa of the brain consistent with metastatic disease (Figure 2).

**Figure 3:**
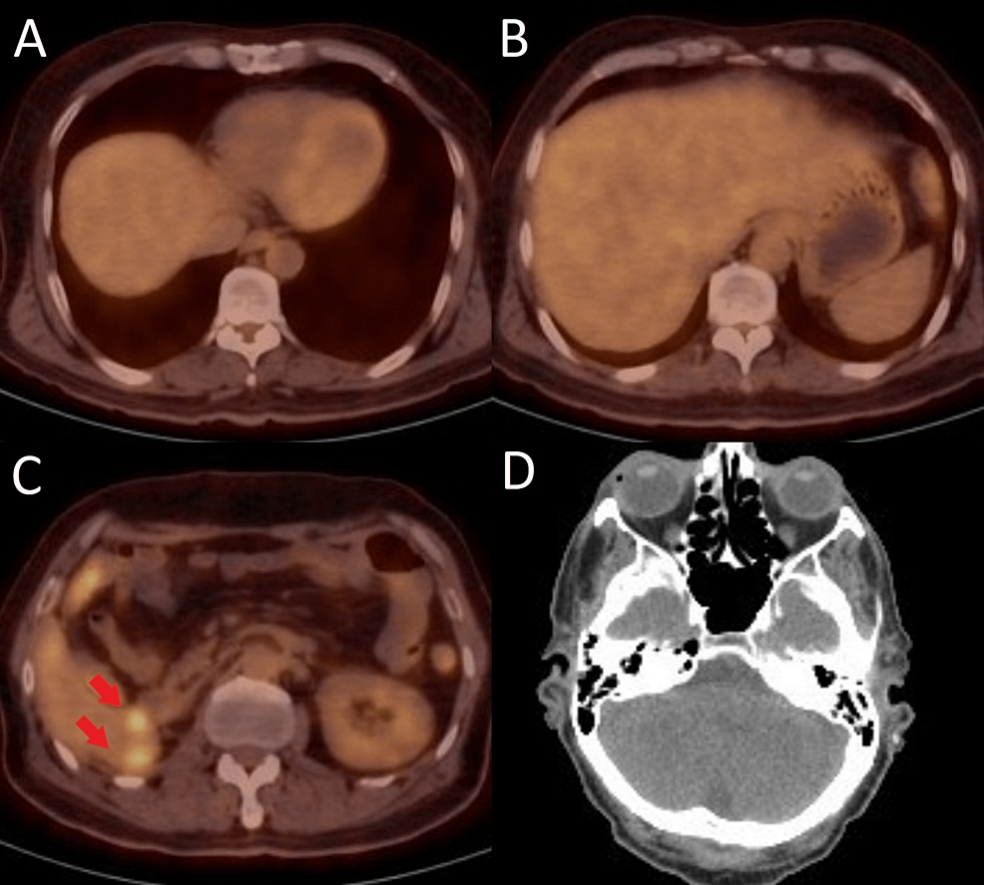
PET/CT images after four months of treatment with nivolumab Positron emission tomography (PET)/CT images showing significant reduction in the size and the number of metastatic lesions following four months of treatment with nivolumab: A) Complete resolution of pleural-based lung metastatic nodule, B) Significant reduction in the size and the number of metastatic lesions in the liver, C) Persistence of a few metastatic lesions in the right hepatic lobe, D) Brain CT scan showing complete radiologic response.

Due to the failure of first-line treatment with sunitinib, treatment was switched to nivolumab 3 mg/kg (240 mg) administered every two weeks. He also received palliative whole-brain radiation therapy (WBRT) to a total dose of 35 Gy delivered during 14 fractions. After four months of treatment with nivolumab, fluorodeoxyglucose (FDG) - positron emission tomography (PET)/CT scan was obtained and it revealed complete resolution of metastatic lesions in the brain and lungs as well as a reduction in the size and the number of lesions in the liver, without the appearance of any new lesions (Figure 3).

**Figure 4:**
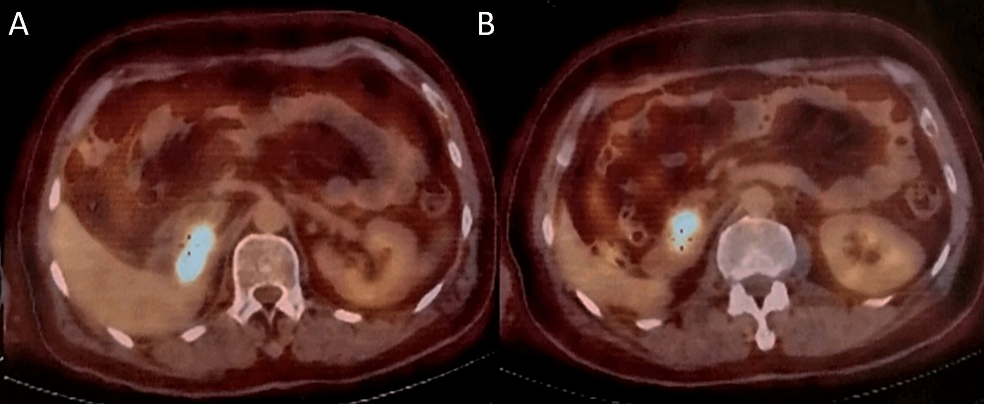
Persistence of a subhepatic mass after six months of treatment with nivolumab, showing activity on FDG-PET/CT scan. FDG: Fluorodeoxyglucose; PET: Positron emission tomography

Hence, he was put on nivolumab for six months and at the end of the treatment he showed complete radiologic response according to Response Evaluation Criteria in Solid Tumors (RECIST) in the lungs and the brain, as well as complete resolution of metastatic lesions in the abdominal wall and liver, except for the persistence of a mass in duodenohepatic region (Figure 4).

**Figure 5:**
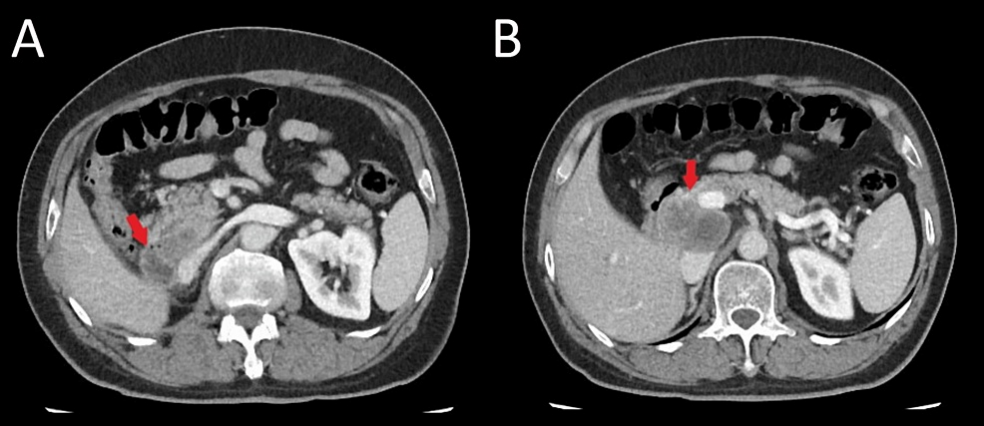
Abdominal CT scan The abdominal CT scan shows a necrotic mass compressing the common bile duct (CBD), with no other pathologic finding elsewhere.

**Figure 6:**
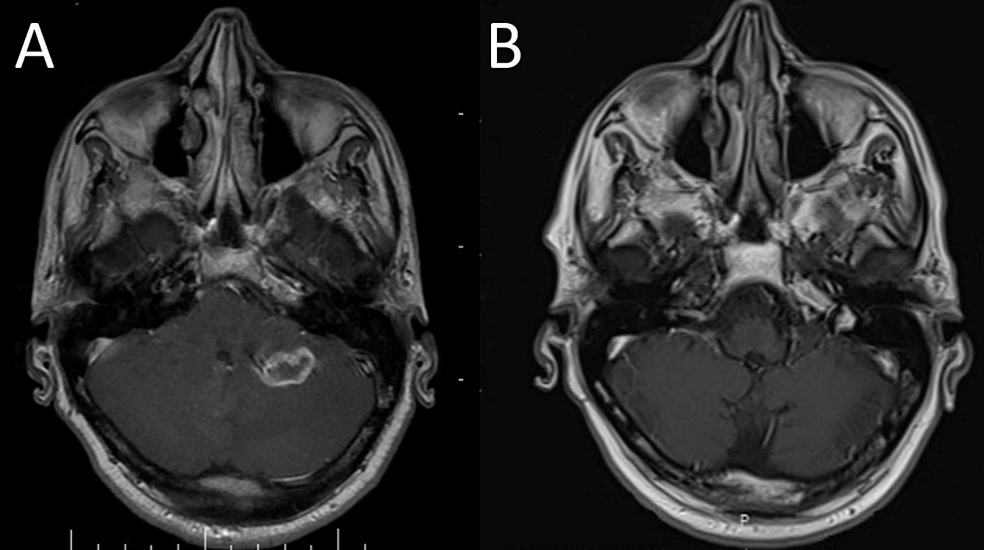
Pre-treatment (A) and post-treatment (B) brain MRI Pre-treatment (A) and post-treatment (B) brain MRI showing complete resolution of metastatic disease in the posterior fossa following the combined treatment with RT and nivolumab (28 months apart).

Unfortunately, nivolumab was discontinued because of financial limitations. He was started on bevacizumab 400 mg every two weeks and was on maintenance bevacizumab for 22 months. On July 2022 the patient became icteric again with high levels of direct bilirubin. A CT scan revealed an 82×50×35 mm mass in the duodenohepatic region compressing the common bile duct (CBD), with no other sign of metastatic lesions in the chest, brain or abdominopelvic CT scans (Figures 5, 6). He is now being managed by a multidisciplinary team to determine the next therapeutic options.

## Discussion

RCC is considered to be radio-resistant and chemo-resistant. The sole class of agents which provided objective responses were cytokine-based treatments with high-dose interleukin-2 (IL-2) and interferon-alpha (IFN-α) [5]. With the introduction of ICIs, the treatment landscape of metastatic RCC continues to rapidly evolve. Nivolumab is a human immunoglobulin G4 antibody targeting the PD-1 receptor, which achieves a durable objective response in many cancers including advanced or metastatic RCC [6]. In the randomized phase III CheckMate 025 study, Motzer et al. showed a superior response rate (RR) with nivolumab compared to everolimus (25% vs. 5%, respectively) and longer median OS in the nivolumab arm (25.0 months vs. 19.6 months, respectively) [2].

GETUG-AFU 26 NIVOREN, was a phase II trial investigating the safety and efficacy of single-agent nivolumab in patients with metastatic RCC. Nivolumab was used as the second line therapy for 63% of patients and as the third line or more for 37%, and the authors concluded that nivolumab showed limited activity against RCC after the failure of other systemic treatments in patients with untreated brain metastasis, based on 12% intracranial RR observed among patients who had not received local therapy, emphasizing the importance of local therapy prior to systemic therapy [7].

Brain metastasis in patients with metastatic RCC generally signifies a poor prognosis. As in other solid cancers, local therapy with either surgery, radiotherapy, or both followed by systemic therapy remains the cornerstone of treatment [3]. The latest guidelines suggest that patients with intermediate or poor risk disease should be offered combination treatment with two ICIs (ie, ipilimumab and nivolumab) or an ICI in combination with a vascular endothelial growth factor receptor tyrosine kinase inhibitor (VEGFR TKI), as the first-line treatment [8,9]. For patients who progress on first-line VEGFR TKI monotherapy (as in our case), the next therapeutic options would be nivolumab or cabozantinib [9]. However, patients with brain metastases are usually excluded from studies, and therefore there are limited data regarding ICIs’ activity in this setting. The backdrop for this exclusion relies on the increased size of ICIs which limits their ability to cross the blood-brain barrier. Brain metastatic patients often need radiation therapy for local control, this combination helps block the immune system’s brakes and to boost the abscopal response rates [4,10]. The abscopal effect was first described in 1953 by Mole. This theory suggests that radiation therapy for solid tumors induces necrosis in tumor cells, and tumor antigens release into the bloodstream. This results in an immune response at a distance from the irradiated volume. It is mostly described in highly immunogenic tumors such as malignant melanoma, RCC, and hepatocellular carcinoma [11].

## Conclusions

We presented a case of metastatic RCC to the brain, lungs, liver, and abdominal wall which showed a significant radiologic response with long-term disease control to treatment with nivolumab, which offers further support for the role of immunotherapy in metastatic RCC. The unique outcome observed in this case was the achievement of complete radiologic response in the brain following treatment with nivolumab and WBRT. The explanation for this desired outcome could be the combination of local therapy (RT) with ICI-based therapy which may have boosted the efficacy of nivolumab on cranial and extracranial lesions (the abscopal effect). The specific challenge with this case was that despite significant radiologic response, the patient could not afford nivolumab for more than six months. Therefore, we were forced to discontinue nivolumab and switch treatment to bevacizumab so that he could afford the treatment. Interestingly, the patient was stable for up to 22 months at which he developed icter due to the subhepatic mass compressing CBD, still with no other sign of progressive disease elsewhere. These results underline the importance of local therapy in combination with ICI-based therapy in RCC patients with brain metastasis, in order to achieve long-term disease control.

## References

[1] Ferlay J, Soerjomataram I, Dikshit R (2015). Cancer incidence and mortality worldwide: sources, methods and major patterns in GLOBOCAN 2012. Int J Cancer.

[2] Motzer RJ, Escudier B, McDermott DF (2015). Nivolumab versus everolimus in advanced renal-cell carcinoma. N Engl J Med.

[3] Brown LC, Desai K, Wei W (2021). Clinical outcomes in patients with metastatic renal cell carcinoma and brain metastasis treated with ipilimumab and nivolumab. J Immunother Cancer.

[4] Kattan J, Rassy EE, Assi T, Bakouny Z, Pavlidis N (2018). A comprehensive review of the role of immune checkpoint inhibitors in brain metastasis of renal cell carcinoma origin. Crit Rev Oncol Hematol.

[5] Massari F, Di Nunno V, Ciccarese C (2017). Adjuvant therapy in renal cell carcinoma. Cancer Treat Rev.

[6] Joseph RW, Chatta G, Vaishampayan U (2017). Nivolumab treatment for advanced renal cell carcinoma: considerations for clinical practice. Urol Oncol.

[7] Courcier J, Dalban C, Laguerre B (2021). Primary renal tumour response in patients treated with nivolumab for metastatic renal cell carcinoma: results from the GETUG-AFU 26 NIVOREN Trial. Eur Urol.

[8] Motzer RJ, Tannir NM, McDermott DF (2018). Nivolumab plus Ipilimumab versus sunitinib in advanced renal-cell carcinoma. N Engl J Med.

[9] Rathmell WK, Rumble RB, Van Veldhuizen PJ (2022). Management of metastatic clear cell renal cell carcinoma: ASCO Guideline. J Clin Oncol.

[10] Fallah J, Ahluwalia MS (2019). The role of immunotherapy in the management of patients with renal cell carcinoma and brain metastases. Ann Transl Med.

[11] Trommer M, Yeo SY, Persigehl T (2019). Abscopal effects in radio-immunotherapy-response analysis of metastatic cancer patients with progressive disease under anti-PD-1 immune checkpoint inhibition. Front Pharmacol.

